# Beyond the Parasite Metaphor: A Morphological Typology of Architectural Intervention

**DOI:** 10.3390/biomimetics11100721

**Published:** 2026-10-10

**Authors:** Anday Türkmen, Selva Başçı

**Affiliations:** Department of Interior Architecture and Environmental Design, Istanbul Gedik University, 34876 Istanbul, Türkiye; selva.basci@gedik.edu.tr

**Keywords:** parasitic architecture, host-parasite analogy, morphological typology, urban densification, adaptive reuse, architectural intervention

## Abstract

Parasitic Architecture represents a radical strategy for urban densification, yet the literature lacks a systematic morphological framework to analyze its physical relationship with the host beyond metaphorical descriptions. Addressing this theoretical gap, this study proposes an action-based analytical typology to classify these interventions. Adopting a purposive, case-based qualitative content analysis, the research examines fifteen documented cases in order to trace how each intervention physically engages its host. The findings identify five distinct morphological attachment strategies: Wedging, Perching, Clinging, Piercing, and Wrapping. These actions are synthesized into a matrix that relates the host interface to the specific spatial strategy of the parasite. Consequently, the findings indicate that these forms can be organized according to their primary morphological action in relation to the host. This study ultimately repositions the concept of the parasite from a critical metaphor into an operational grammar of architectural intervention, offering an analytical tool with potential applications in adaptive reuse and urban design.

## 1. Introduction

The theoretical and historical foundations of design disciplines, such as Architecture, Interior Architecture, and Urban Design, are built upon a fundamental principle focused on meeting human spatial needs, providing stable living environments, and enhancing the quality of these environments. The normative principle of these disciplines is to optimize spatial organization in a manner that is functionally, structurally, and aesthetically permanent and complete. These micro-scale design objectives integrate with the broader societal ideals aimed at by Urban and Regional Planning at the macro-scale, such as steady growth, clarity of zoning regulations, defined property boundaries, and a predictable urban fabric. The structural order and defined plots envisioned by planning gain functionality precisely through this permanent, static, and completed nature of architectural objects.

However, in contemporary urban practices, a paradoxical orientation contradicting these fundamental functional goals and the principle of permanence is observed. The increasing density of cities, the proliferation of residual urban spaces, and the diversification of instantaneous or temporary spatial needs have led to the spread of practices that challenge architecture’s nature of producing permanent and complete solutions. This approach, conceptualized as *Parasitic Architecture*, shifts the role of architecture from producing a static object to a dynamic act of intervention. These interventions intentionally occupy or cling to existing buildings and residual urban voids, which planners and traditional architecture envision as *completed* and *whole*. This situation is not merely a technical issue of an addition; it provokes a profound interrogation of the core codes of architecture such as *permanence, ownership*, and *formal integrity*, as well as the relationship established with the existing building stock, thereby creating a critical and interdisciplinary field of debate.

Despite the popularity of the *parasitic architecture* phenomenon in contemporary architectural discourse, an examination of the academic and critical literature points to a specific gap in this field. The existing literature predominantly addresses the concept through philosophical, socio-political, or theoretical dimensions, or focuses on isolated case studies. In addition, several studies offer typological frameworks for architectural additions and interventions, ranging from studies of new additions to built heritage [1,2] and conservation-oriented classifications of historic buildings [3,4] to operational and processual frameworks [5,6] and conceptual or design-based approaches to parasitic architecture [7,8,9], yet these frameworks treat intervention types mainly through functional, formal, or processual lenses. While these approaches offer a valuable ground for understanding the conceptual infrastructure and singular examples of the phenomenon, they leave unresolved the systematic classification and comparison of these interventions from a morphological perspective. In the current state, physically distinct actions, such as the act of being situated on a building’s roof versus the act of occupying the void between two buildings, are frequently aggregated under broad labels without articulating how each intervention physically engages its host. This lack of morphological classification causes *parasitic architecture* to remain mostly as a conceptual metaphor rather than an analytical tool defining specific morphological intervention forms. This situation makes it difficult to compare designs and existing applications on an objective basis, to analyze them, and to use them methodologically in design education. In this context, the fundamental problem of the research is the absence of a comprehensive and analytical morphological typology based on the physical actions through which parasitic architectural interventions engage their host structures.

In direct response to the problem defined above, the fundamental objective of this study is to develop a systematic typology model that classifies parasitic architectural interventions based on the physical, morphological, and structural relationship forms they establish with the host structure. By accepting the existing conceptual discussions within the literature as a foundational ground, this work explicitly focuses on how these interventions are morphologically constructed. In this context, the research seeks a comprehensive answer to the following Research Question (RQ):

RQ: How can parasitic architectural interventions be systematically classified according to their physical relationship with the host structure?

The search for an answer to this inquiry is of paramount importance, as it holds the potential to elevate the term parasite from a vague metaphor to a defined academic concept and a functional analytical tool equipped with clear criteria. The typology to be developed aims to offer architects, urban planners, and students the capability to analyze and compare existing or proposed parasitic interventions on an objective basis, thereby fulfilling the dual goal of bridging the gap in the academic literature and making a distinct methodological contribution to design education.

The novelty of the proposed typology can be stated against the two families of classification that it engages. Existing classifications of additions group interventions according to static location and geometric relation; they describe where an intervention is positioned, not how it physically engages its host. A second family classifies interventions by the action performed rather than by location: it decomposes the incremental production of urban space into discrete operations such as extending, attaching, replacing, dividing, infilling, and connecting, proposes a relational vocabulary of building over, within, alongside, and around, or classifies parasitic forms according to the nature of the host relationship. These frameworks name the operation, but they do not specify the host interface through which the operation is physically realized, nor do they connect it to a structural principle and a spatial outcome. The typology proposed here is organized by the physical action through which an intervention engages its host and, for each action, specifies the host interface it requires, the structural principle it implies, and the spatial strategy it produces. Its contribution is therefore not a new vocabulary of operations but the operationalization of the physical act of attachment, converting the static and action-based classifications into a single systematic instrument through which parasitic interventions become comparable and, consequently, design-operable.

While this study offers a significant typological contribution to the field by analyzing the parasitic architecture phenomenon through the axis of physical attachment actions, or morphology, to the host structure, it must be explicitly noted that the study’s findings and the developed morphological typology are subject to specific theoretical and methodological limitations. The primary dataset, or corpus, of the study is constituted by existing secondary sources regarding parasitic applications, which were utilized to develop the proposed typology. These sources are limited to documented examples found in peer-reviewed academic literature, such as articles and book chapters, professional architecture and design publications, and reputable media sources. This situation serves as a significant factor affecting the external validity and transferability of the research, creating a constraint on the generalizability of the findings to examples not yet documented in the literature or those existing in different geographical or cultural contexts, such as non-Western cities or vernacular and spontaneous interventions. As a natural consequence of this methodological choice, the research does not include primary data collection processes, such as conducting new field observations in a specific urban context, carrying out on-site structural analyses of interventions, or holding in-depth interviews with the designers or users of these designs.

Consequently, the study’s findings and the developed typology are grounded in the qualitative content analysis of a purposefully selected corpus of documented cases. Finally, as a theoretical limitation, the research has deliberately focused its scope on the physical and morphological attachment forms of the interventions. Other significant dimensions, such as detailed structural engineering analyses, material performances, implementation costs, or the long-term sociological effects on the users of the host structure, have been excluded from the analytical framework of this study. Accordingly, the Structural Principle column of the proposed analytical matrix and the related descriptions in Section 4 characterize the attachment logic of each category as it is described in the cited documentation and evident in the visual material of the cases; they do not constitute engineering verifications and do not claim to certify the structural performance of the interventions. In addition, formal inter-coder reliability measures and external peer debriefing were not part of the study design; the classification was developed jointly by the authors, and its independent application by other researchers, with an associated agreement measure, is proposed as a direction for future research.

## 2. Theoretical Framework

The term parasite biologically implies vital dependency on a host, yet its transposition to spatial theory is grounded in a deeper philosophical framework. Michel Serres [10] re-conceptualized the parasite not merely as a consumer, but as a catalyst or noise that interrupts a system to increase its complexity. This philosophical ground was carried into architectural theory by Marini [11], who follows Serres’s Le Parasite to read the parasite simultaneously as a biological metaphor, a social figure, and an intruder of the linguistic structure, deploying these three interpretations as a frame for distinguishing the tendencies of contemporary parasitic architecture. This perspective gained early architectural expression through Oswald Mathias Ungers [12], who defined parasitic architecture in *City Metaphors* as an informal organization, interpreting the attachment to infrastructure as a manifestation of survival instincts. Contemporary studies, such as Baroš and Katunský [13], reframe this approach within biomimetic design, highlighting its potential to generate architectural diversity, yet they equally insist on the duality of the parasitic condition: capable of creating diversity as readily as causing extinction, and of neutering the host or accelerating its growth, the parasite resists any naïve one-to-one transfer into architectural design.

Current morphogenetic discourse centers on the transfer of these biological strategies into architectural practice. To grasp the operational logic of parasitic structures, which are regarded as a speculative method for architecture to sustain its existence within chaotic urban conditions, it is requisite to first focus on their survival strategies and stages of evolutionary adaptation within the biological realm.

**Figure 1:**
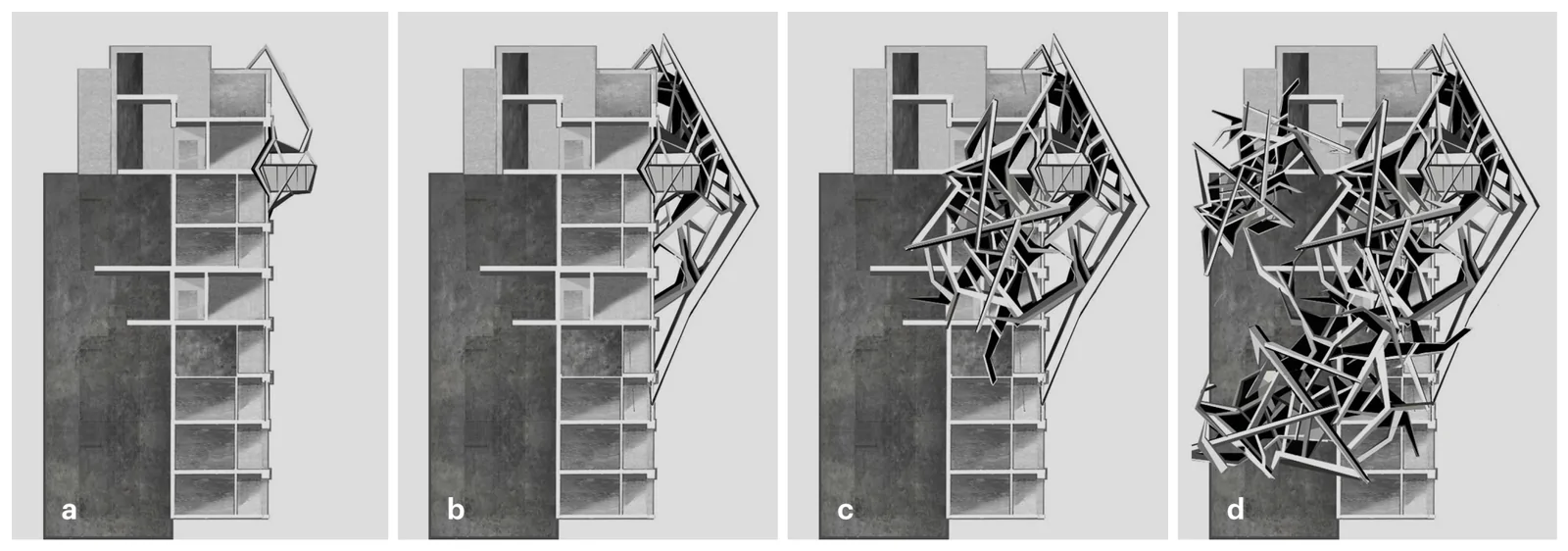
The parasitic architecture and the process of host adaptation [14]. (**a**) Phase I: Encounter; (**b**) Phase II: Attachment; (**c**) Phase III: Establishment; (**d**) Phase IV: Exploitation.

This biological adaptation process finds a spatial counterpart upon its transfer to the architectural realm. Parasitic design, referencing the ecological relationship between the parasite and its host, gains a symbiotic meaning at the architectural scale; the reflexes enabling the parasite to adapt to the host through an evolutionary process transform into structural morphological actions (Figure 1).

The four phases presented in Figure 1 are named after the filter concept of Combes, who conceptualized the host–parasite interaction as a succession of filters: the encounter filter, which determines the probability of contact between the parasite and the host, and the compatibility filter, which determines the probability that the parasite and the host can live together [15]. The stepwise characterization of the infection process in the parasitological literature complements this framework: the parasite must encounter the host, attach to the host tissue and cross the host epithelium, establish a durable infection within the host, and eventually proliferate to produce transmission stages [16]. Condensed into the phases of Encounter, Attachment, Establishment, and Exploitation, these concepts indicate the temporal dimension of the parasitic relationship.

However, within architectural literature, while every parasitic structure is technically an extension or addition, not every addition can be characterized as a parasite. Arabulan and Lank [17] draw attention to the conceptual ambiguity in the literature, stating that labeling every installation or structural element as a parasite leads to misinformation. Therefore, to define the conceptual boundaries of this study and to substantiate the sample selection, the typical characteristics distinguishing conventional additions from parasitic architecture have been synthesized comparatively in Table 1, in light of discussions in the literature [18,19,20]. It is clarified that the four criteria are understood as prototypical features of the parasitic condition rather than as necessary conditions. A case is characterized as parasitic when it predominantly exhibits these features, and each criterion may be satisfied to a different degree. This graduated understanding reflects the parasitological distinction between obligate and facultative parasites, and between temporary and permanent parasites, which describe a spectrum of host dependence rather than a binary condition [21,22].

Examination of the historical roots of parasitic architecture highlights the radical movements of the 1960s. Archigram and the Japanese Metabolists envisioned the city as a dynamic metabolism rather than a finished composition, establishing the early logic of the parasite through plug-in capsules attached to existing infrastructure. However, a more critical and political reading was advanced by Lebbeus Woods [23]. In his proposals for war-torn cities, Woods framed the parasite not as a tool for healing, but as a form of architectural resistance against established authority. For Woods, the parasite represents the new clinging to the old, refusing to accept its rules.

Existing literature on *parasitic architecture* predominantly centers on conceptual definitions and isolated case studies, and although recent work has begun to reframe the parasite as a domesticated and symbiotic mechanism within incremental housing [24], a systematic classification of its morphological actions remains absent. Furthermore, technical classifications of additions generally categorize interventions via static parameters such as location and geometry [25,26]. However, these classifications leave unaddressed the physical action through which the intervention engages its host. The same limitation applies to the action-based frameworks that classify interventions by the operation performed rather than by location [5,6,7]: these frameworks identify the operation but do not specify the host interface through which it is realized, and they are not formulated as a morphological typology of attachment. Comparable constraints recur in adjacent lines of inquiry. Assessment models for constructional performance in extensions to historical buildings [27], adaptive reuse frameworks that define addition as a general strategy [28], layered accounts of the physical constitution of buildings [29], and palimpsest-based theories of remodeling [30] each address intervention through a distinct analytical register, whether performance, function, or compositional logic. What these frameworks have in common is that the physical action performed on the host does not serve as the organizing criterion.

At this juncture, contemporary design discourse strengthens the theoretical ground by shifting the ontological status of architectural form from a *static object* to a *processual and systemic construct*. This framework necessitates redefining *parasitic architecture* not as a frozen addition, but as a dynamic *morphological intervention process* responsive to the host. Positioning the proposed typology as a morphological typology therefore requires anchoring it within the foundational concepts of urban morphology. If parasitic architecture is understood as a processual intervention rather than a static addition, its action-based classification must connect with the discipline studying how urban form is transformed over time. The primary foundation rests on the Conzenian tradition, which analyzes urban tissue through street pattern, plot, and building [31,32]. This framework yields two key implications: first, the building scale occupied by the parasite functions as an embedded node within plot and street contexts; second, the incremental infilling described by the burgage cycle provides a morphological precedent for residual space occupation at the urban scale. Complementing this, the typological tradition from Muratori to Caniggia grounds the reading in the concepts of type, tissue, and transformation [33,34]. Here, architectural type is understood not as a static object, but as a generative matrix for ongoing spatial evolution. Viewed through this lens, the parasitic addition appears as a transformative phase within the evolution of urban tissue. Moudon brought these traditions together into a unified typomorphological perspective [35,36], while recent empirical research indicates that incremental volumetric additions represent a standard urban growth process. The proposed typology is thus positioned where historico-geographical analysis (street-plot-building) meets processual typological transformation [37,38,39], establishing the disciplinary ground for a morphological typology of architectural intervention. Currently, the literature lacks a typology that classifies these interventions according to their physical relationship with the host; this study aims to fill this theoretical gap.

## 3. Methods

This section comprehensively presents the methodological framework adopted to answer the research question (RQ). The method section is structured around the research model that guided the study’s design, the selection process and justification for the dataset, or corpus, included in the analysis, the data collection procedures employed, and the data analysis strategy utilized to synthesize the findings. The research is based entirely on publicly available and open-access secondary sources. At all stages of the research, the principles of scientific research and publication ethics were rigorously adhered to, particularly regarding the transparent and accurate citation of all references used.

This research adopts a purposive, case-based qualitative analysis model to analyze the parasitic architecture phenomenon and develop an original morphological typology. Unlike a traditional literature review, this approach treats the literature, comprising academic publications, professional portals, and critical analyses, not as the object of the review itself, but as the primary source from which documented cases are identified. The search was purposive rather than exhaustive: it aimed to locate documented cases meeting the predefined inclusion criteria described below, and the selection, extraction, and analysis procedures are described in this section.

The universe of this research was constituted by publicly documented architectural intervention examples that exhibit practices of Parasitic Architecture and are accessible through the academic databases and design portals searched in this study. From this universe, the dataset, or corpus, of the study was selected using the purposive sampling technique, and specifically through the method of criterion sampling. As a methodological constraint of this documentation-based design, all fifteen cases included in the corpus are located in European and North American contexts; the study does not claim to cover parasitic interventions in other geographical or cultural contexts. In accordance with this methodological approach, only those examples that met a set of specific, pre-defined criteria were included in the final dataset. These criteria stipulated two primary conditions: first, the case must possess the specific attributes (e.g., dependency, provocation) of the conceptual parasite definition, which was distinguished from the conventional addition within the theoretical framework of the study; and second, it must possess sufficient visual data (such as photographs, technical drawings, or diagrams) and/or textual data (such as architectural descriptions or critical reviews) to enable a detailed morphological analysis regarding its form of physical attachment to the host structure. Consistent with this purposive, case-based approach, the data collection was carried out as a document review in several steps, as described in the remainder of this section. This process involved the systematic use of predefined keywords and their semantic derivatives, specifically parasite architecture, architectural additions, urban interventions, rooftop extensions, and infill architecture. These terms were utilized to search within major interdisciplinary academic databases (e.g., Scopus, Web of Science, and Google Scholar) as well as within prominent design portals that document professional reflections and case studies regarding the topic (e.g., ArchDaily, Dezeen). The documents retrieved from this comprehensive search (including research articles, architectural case studies, and critical analyses) that met the predefined inclusion criteria were subsequently compiled to form the final dataset, or corpus, comprising fifteen documented cases (see Table A1 for the complete list), for the analysis.

The analysis of the collected qualitative data, comprising both visual and textual documents, was conducted using the qualitative content analysis technique in a deductive manner. The conceptual framework of the study, including the defining characteristics of the host–parasite relationship established in Table 1, served as the analytical starting point against which the case data were examined. As the initial deductive step of the analysis, a customized data extraction form was designed and utilized to process the data systematically. This form was structured directly to address the primary objective of the research: understanding the physical relationship forms established by parasitic structures with the host. For each case in the final dataset, or corpus, all relevant data describing the morphological, physical, and structural relationship established by the structure with the host, such as how it is suspended, where it sits, or what it is wedged between, were recorded into this form from the source texts and visuals. Following this deductive data extraction process, the recorded data were coded according to the primary morphological action each case performs with respect to its host, for example, “attaching by wedging between two buildings”, “connecting by perching on the roof”, or “attaching by clinging to the facade”, and the resulting codes were cross-compared across the corpus to determine whether the cases could be consistently grouped under the categories of the framework. In the final synthesis stage of the analysis, the verified categories and their defining codes were interrelated and subjected to cross-comparison. It is acknowledged that the typology constitutes a deductively derived analytical framework verified against a purposefully selected corpus of documented cases, rather than a set of categories claimed to emerge from the data.

Classification was based on the primary morphological action performed by the intervention in relation to its host. Each case was assigned to a single category according to its primary action, defined as the action that most assertively and visually dominates the parasite-host relationship, as documented in the cited sources and observed in the visual material. When an intervention exhibited features associated with more than one action, it was classified according to its primary action; secondary features are noted in the case descriptions but do not determine the classification. All fifteen cases of the corpus were successfully assigned to one of the five categories, with three cases per category. No case within the corpus resisted classification. The classification was carried out jointly by the authors through consensus-based coding, whereby disagreements were resolved through joint discussion. Because the analysis was applied to the complete corpus of fifteen cases rather than to an open-ended sample, the conventional criterion of saturation is replaced here by the exhaustive coverage of the corpus; the five categories are accordingly considered exhaustive with respect to the analyzed corpus, and their coverage beyond this corpus is not claimed. The framework was additionally tested against negative cases, namely the Keret House, where the mechanically expected load path of the occupied gap is absent, and the Royal Ontario Museum, which combines features of more than one action.

## 4. Results

This section presents the analytical resolution of the final dataset, or corpus, which comprises all fifteen documented cases analyzed in this study, and which was compiled according to the conceptual criteria previously defined in the study. As detailed in the methodology section, the deductive, framework-based qualitative content analysis approach was utilized as the primary structuring tool for this section. This approach aims to overcome the problem of Parasitic Architecture practices being addressed in the literature predominantly in a metaphorical or morphologically unsystematic, scattered manner. Therefore, the findings presented below directly answer the fundamental research question (RQ) of the study and present these synthesized findings as the final and original contribution of the research: An analytical typology based on morphological attachment actions.

The fundamental research question (RQ) aimed to decipher how parasitic interventions physically attach to host structures. In this context, the framework-based qualitative content analysis applied to the cases in the corpus indicated that these interventions, which are often presented in the literature in a scattered manner (e.g., as “rooftop extension” or “interstitial structure”), are consistently describable in terms of specific physical attachment actions. Furthermore, the analysis suggests that the design interventions used are systematically repeated in line with specific morphological actions and can be grouped under five main analytical categories, each represented by three of the fifteen cases. These categories represent the fundamental morphological attachment actions that parasitic designs utilize to physically connect to a host structure. At the same time, it is acknowledged that three cases per category constitute a modest empirical base. The typology is therefore presented as an analytical framework derived from a purposively selected corpus of documented cases, rather than a statistically exhaustive classification of parasitic interventions.

### 4.1. Wedging

**Figure 2:**
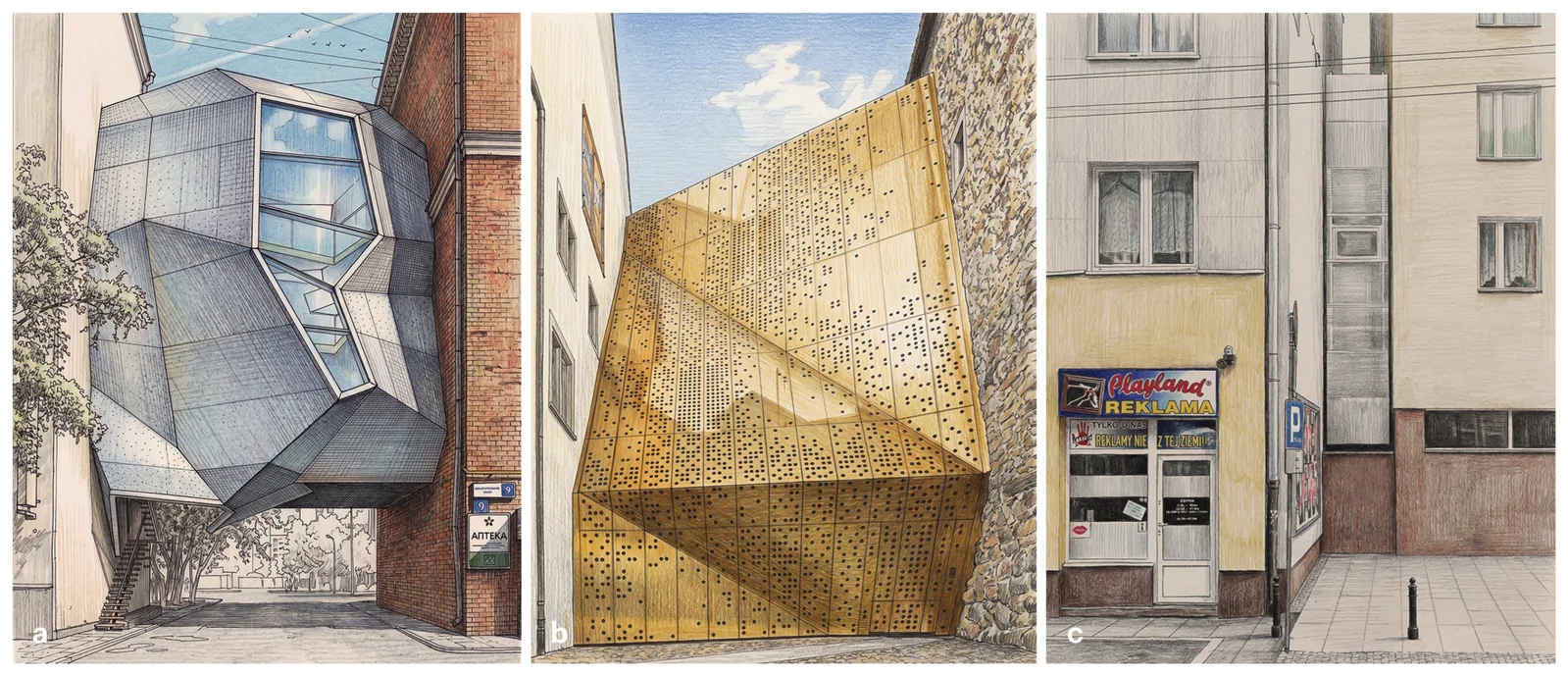
The Wedging Morphological Action: (**a**) Parasite Office, Moscow/Russia [40]; (**b**) Stadtmuseum, Rapperswil-Jona/Switzerland [41]; (**c**) The Keret House, Warsaw/Poland [42] (redrawn by the authors based on [40,41,42]).

The first distinct morphological action identified within the analyzed dataset has been categorized as Wedging. The fundamental spatial strategy defining this category is predicated on the physical occupation of the interstitial or gap spaces situated between two or more existing host structures. The primary operative technique of this specific action involves the parasitic structure intentionally utilizing the lateral vertical planes of the hosts, particularly blind facades, to establish both physical attachment and spatial definition. Consequently, this morphological action effectively transforms an urban volume previously coded as residual or negative space into a physically usable and positive architectural environment. Within the literature, these interventions typically manifest as infill structures that appear to be suspended, squeezed, or inserted like a wedge between larger, permanent urban masses (Figure 2).

The cases documented in Figure 2 are consistent with the reading that this category functions not merely as a static location between buildings, as often depicted, but as a unique strategy of spatial dependency. These examples indicate that this action is defined primarily by the occupation of the gap between the lateral surfaces of the host. It is acknowledged that the structural mechanisms employed within this category vary between cases and are reported only as described in the cited sources. These mechanisms range from completely suspended systems (such as the Parasite Office) to structurally independent frames (such as the Keret House). Regardless of these variations, the intervention remains fundamentally dependent on the opposing vertical walls for its spatial definition. Even when the host surfaces do not provide load-bearing support, they set the physical boundaries and the contextual rationale of the design. The form and volume of the Keret House, for instance, are directly shaped by the negative void generated by the adjacent buildings, and its parasitic identity is likewise defined by this void. The morphology of the examined examples indicates that the parasite avoids imposing an ideal form, instead adopting a fluid and morphing strategy to occupy even the most irregular urban gaps, as seen in Stadtmuseum. In this context, Wedging transforms negative inert volumes into positive usable spaces. It also functions as a remedial urban infill tactic that physically stitches interruptions in the building stock and repairs the urban silhouette.

### 4.2. Perching

**Figure 3:**
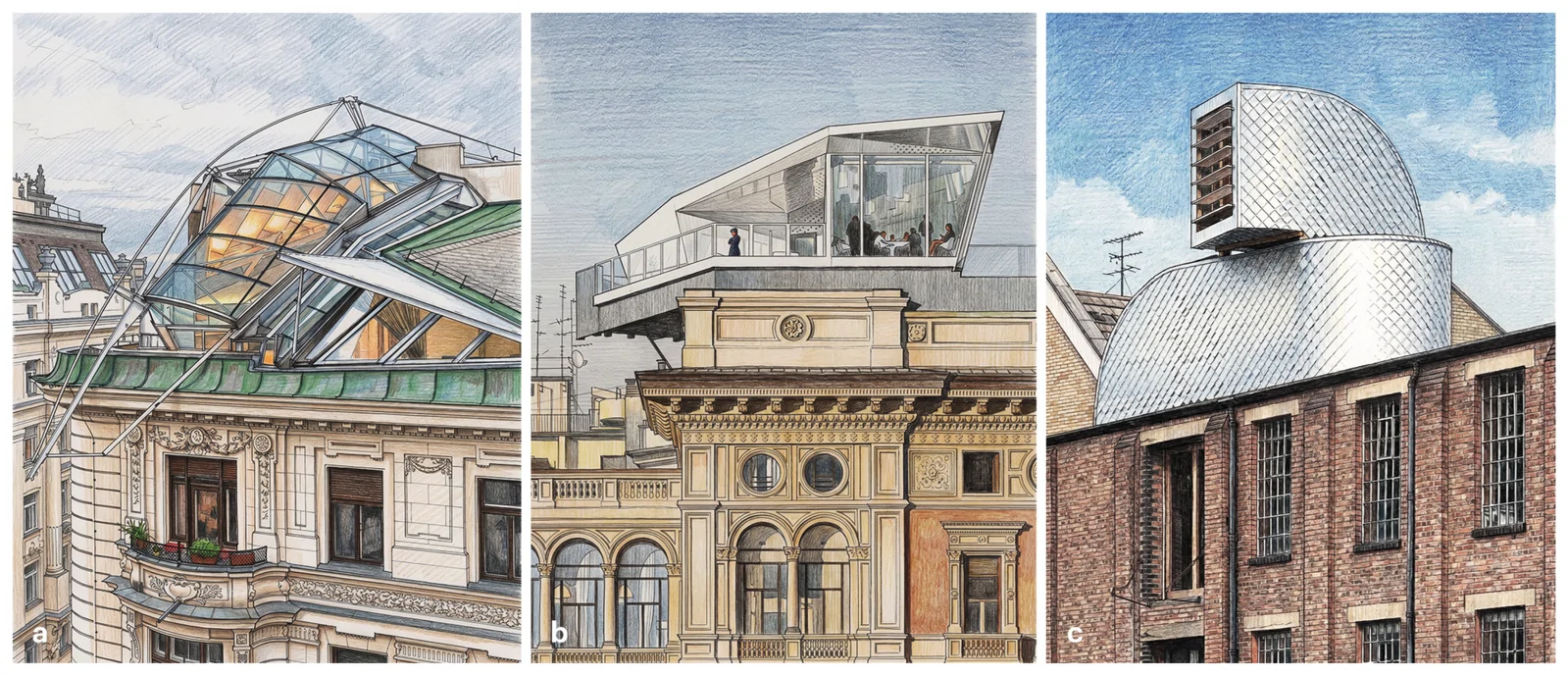
The Perching Morphological Action: (**a**) Rooftop Remodeling Falkestrasse, Wien/Austria [43]; (**b**) The Cube Restaurant, Milan/Italy [44]; (**c**) H-VAC Antepavilion, London/UK [45] (redrawn by the authors based on [43,44,45]).

The second distinct morphological attachment mode identified within the dataset is categorized as Perching. The fundamental strategy defining this category is predicated on the superimposition of the parasitic structure directly atop the host structure, specifically occupying its roof or uppermost horizontal plane. The primary operative technique of this action involves direct vertical bearing onto the existing structure of the host, which effectively functions as a pedestal or platform for the intervention. This relationship enables the parasite to ascend beyond the existing building mass, thereby exploiting the host’s vantage point, iconic location, or elevation to establish a provocative new stratum within the urban silhouette. In the literature, these interventions typically manifest as lightweight additions that appear to have landed on existing rooftops, often remaining intentionally alien to the host in terms of form or materiality (Figure 3).

The cases documented in Figure 3 are consistent with the reading that the Perching category operates not merely as a traditional vertical extension, but as a strategy of adding an independent and alien object atop an existing mass. The fundamental morphological pattern indicated by the visual material is that the parasite transforms the roof into a display platform by establishing maximum contrast rather than formally integrating with the host structure. The Falkestrasse example (a) represents the most assertive and visually dominant form of this action; the parasite does not mimic the host’s neoclassical order but radically disrupts the existing silhouette by perching upon it with a deconstructivist form. The Cube (b) example suggests the lightness of the action and indicates how the parasite exploits not only the host’s structure but also its view and prestige. The H-VAC Antepavilion (c) introduces a distinct morphological tactic to the Perching typology; here, the parasitic intervention itself is shaped as an inert technical volume (a ventilation duct) placed on the host roof. The duct-like form constitutes the intervention, allowing it to pass as a harmless infrastructural element and to bypass planning restrictions. This situation indicates that the act of perching is a flexible superposition strategy that can operate not only as an overt addition but also through a mimetic disguise.

### 4.3. Clinging

**Figure 4:**
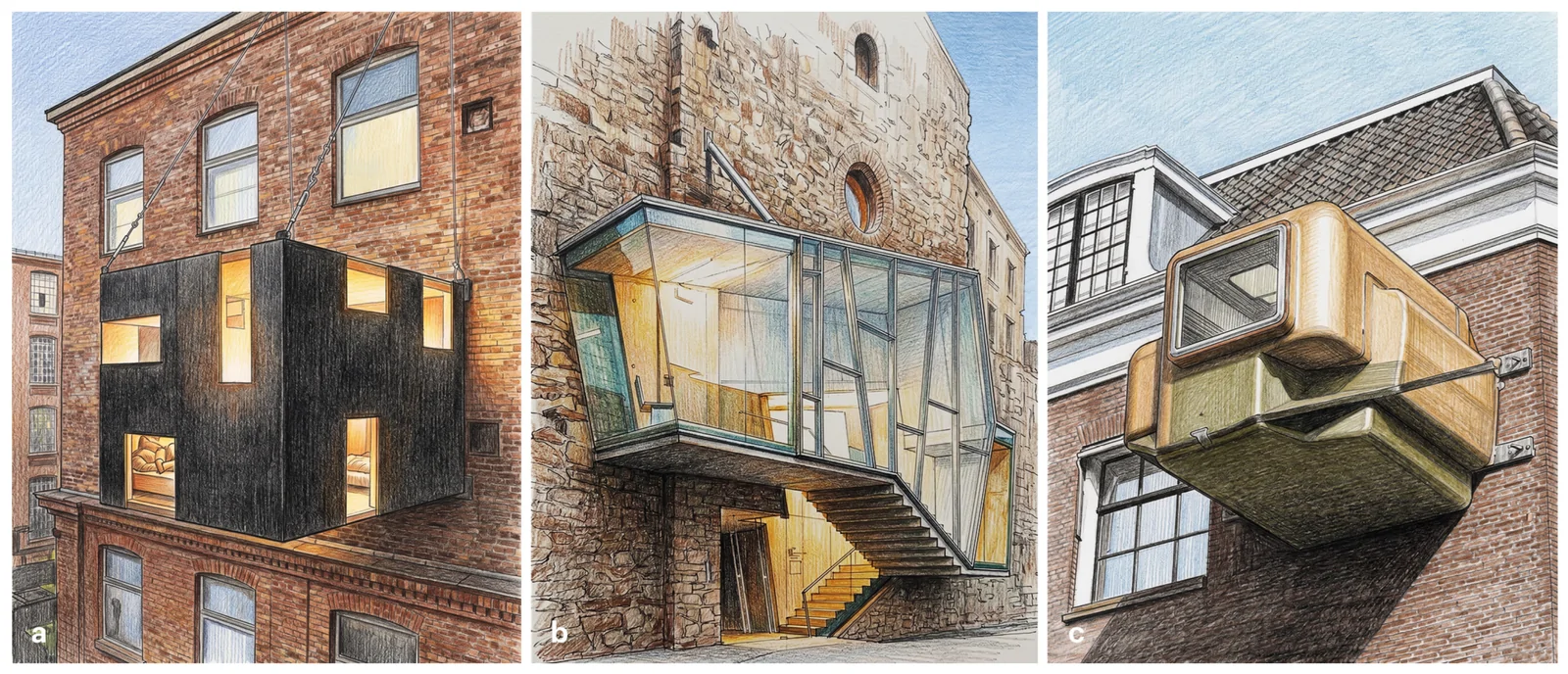
The Clinging Morphological Action: (**a**) Rucksack House, Leipzig/Germany [46]; (**b**) Convent de Sant Francesc, Santpedor/Spain [47]; (**c**) Clip-On Centraal Museum, Utrecht/Netherlands [48] (Redrawn by the authors based on [46,47,48]).

The third morphological attachment mode identified within the dataset is categorized as Clinging. The fundamental spatial strategy defining this category is predicated on the parasitic structure suspending itself from a vertical surface of the host, such as a blind facade, a retaining wall, or an infrastructural pier. Unlike the vertical resting logic of Perching, the primary operative technique here involves a lateral attachment mechanism, achieved through cantilevered or anchored suspension systems fixed directly into the host’s flank. This action effectively activates underutilized vertical planes, generating floating volumes that often maintain no direct physical contact with the ground. In the literature, these interventions typically manifest as prosthetic additions, such as modular cabins attached like a backpack or cantilevered masses projecting outwardly from the host’s envelope (Figure 4).

The cases documented in Figure 4 are consistent with the reading that the Clinging category is a strategy engaging parasitic architecture in a radical struggle with gravity to produce groundless volumes within urban space. The visual material indicates that this action operates not merely as an addition method but as a protrusion extending the host structure’s boundaries outwardly. The Rucksack House (a) represents the most literal, nomadic form of this strategy; the parasite utilizes the host facade merely as a temporary suspension surface, transforming architecture into a portable object. Conversely, the Convent de Sant Francesc (b) exhibits the remedial potential of the action; here, the parasite clings not to a flat wall but to a ruined edge, completing and healing the historic wound. Clip-On (c) displays the most assertive formal expression; adhering to a historic brick facade, this amorphous, contrasting mass acts as a foreign organism intentionally violating the host’s gravity. Collectively, these examples suggest the operational capacity of the Clinging action to transform inert vertical surfaces into active living spaces.

### 4.4. Piercing

**Figure 5:**
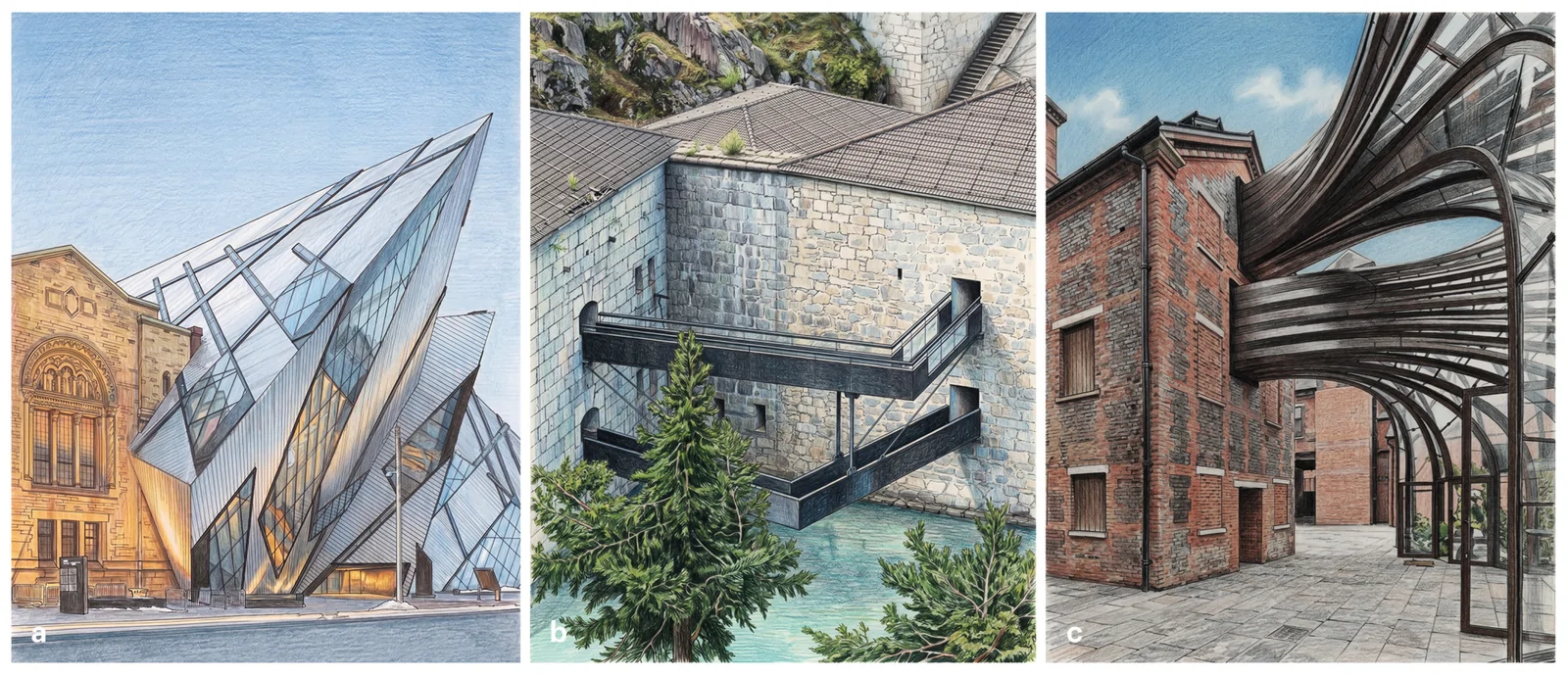
The Piercing Morphological Action (**a**) Royal Ontario Museum, Toronto/Canada [49]; (**b**) The Fortress of Franzensfeste, Fortezza/Italy [50]; (**c**) Bombay Sapphire Distillery, Hampshire/UK [51] (redrawn by the authors based on [49,50,51]).

The fourth distinct morphological action identified within the dataset is categorized as Piercing. The fundamental strategy defining this category entails the parasitic structure physically penetrating, infiltrating, or embedding itself into the existing envelope (facade, roof, floor) or volumetric mass of the host. The primary operative technique of this action is the intentional disruption of the host’s existing morphological integrity. This intervention establishes a tense and inseparable spatial relationship wherein the parasite enters the interior of the host and physically fuses with it while retaining its distinct identity. Often representing the most provocative form of morphological intervention, this action redefines the host’s spatial organization, interrupts its continuity, or generates new internal circulation and spaces. In the literature, these interventions typically manifest as new structures passing through existing buildings or distinct masses cutting through historic shells (Figure 5).

The cases documented in Figure 5 are consistent with the reading that the Piercing category constitutes a specific boundary violation that fundamentally redefines the host structure’s limits and integrity, moving far beyond a mere addition. The visual material indicates that this relationship operates not as passive surface contact but as an active process of penetration and fusion. The Royal Ontario Museum (a) represents the most dramatic instance framed through collision; here, the crystal mass does not merely lean against the facade but actively slices into it, creating a complex interlocking. Although the crystal volume also rests upon and partly envelops parts of the host volume, the primary action of the intervention is determined as Piercing, since the penetration and slicing of the host envelope constitutes the most assertive and visually dominant morphological relationship of the case. Fortezza (b) displays surgical precision; the parasite physically pierces through thick historic walls to enable an otherwise impossible circulation path. Bombay Sapphire Distillery (c) presents an organic version; here, the parasite pierces the existing shell as a fluid architectural form overflowing from within. Collectively, these examples suggest that the parasite establishes its existence by intentionally violating and redefining the host’s physical boundaries and formal logic.

### 4.5. Wrapping

**Figure 6:**
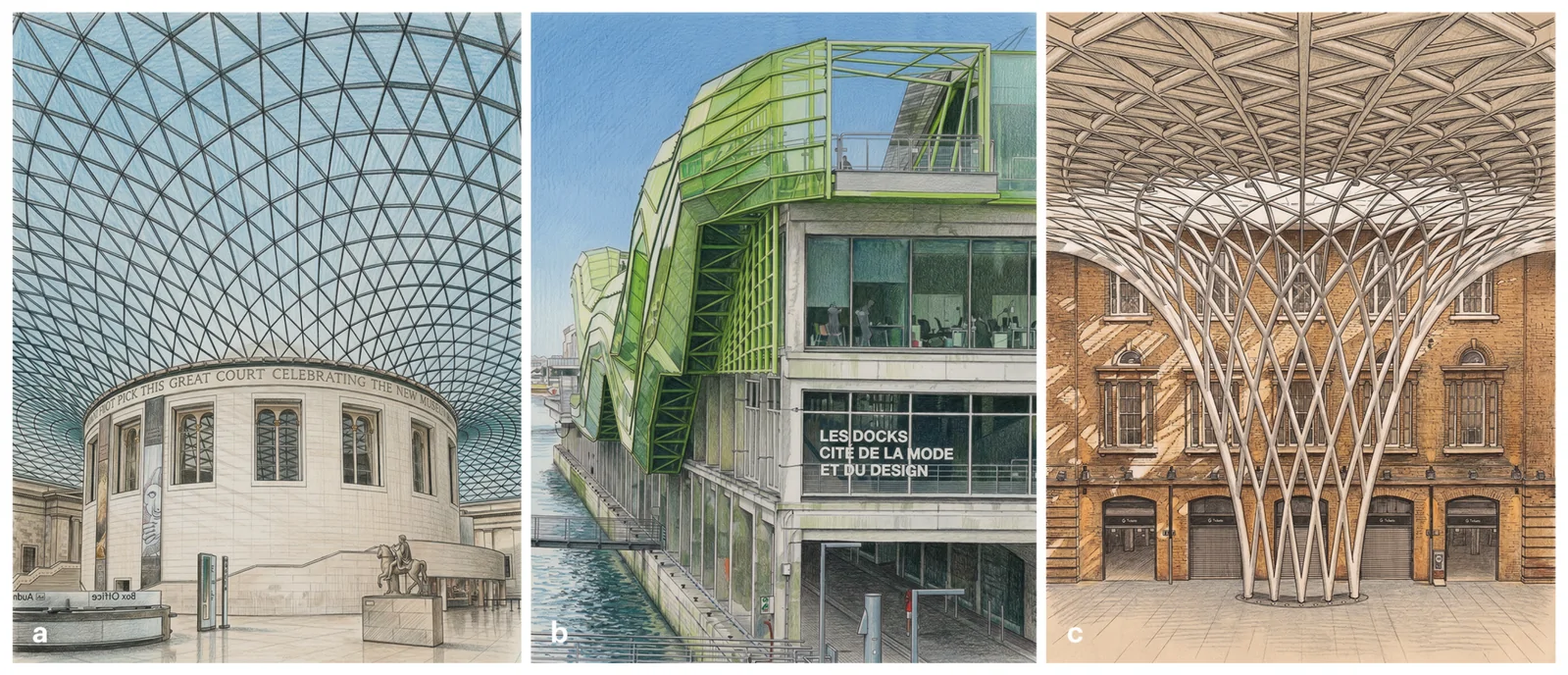
The Wrapping Morphological Action (**a**) The Great Court, London/UK [52]; (**b**) Cité of Design and Fashion, Paris/France [53]; (**c**) King’s Cross Station, London/UK [54] (redrawn by the authors based on [52,53,54]).

The final morphological action analyzed in this study is identified as Wrapping. The fundamental strategy of this category is based on the parasitic structure surrounding, enclosing, or being added as a new skin or envelope to an existing host structure or an urban void, such as a courtyard. The primary technique of this specific action is the creation of a new external or internal enclosure by the parasite wrapping around the host. This new shell either attaches to the existing host or contains the host through an independent load-bearing system. The latter situation represents the facultative end of the dependence spectrum: the parasite does not rely on the host for structural survival, yet its spatial existence remains entirely conditioned by the host’s volume, position, and void, which it requires in order to realize its wrapping action. This reading is consistent with Table 1, in which dependence is defined as a prototypical rather than an absolute trait of the parasitic condition. This action fundamentally redefines the host’s relationship with the external world in climatic, visual, and functional terms; for instance, it can transform a residual courtyard into an interior space or grant an entirely new identity to the facade of an old building. In the literature, these interventions manifest as wide-span glass or steel canopies covering historic courtyards or as second skins that completely coat an existing building (Figure 6).

The cases documented in Figure 6 are consistent with the reading that the Wrapping category constitutes as an action of generating encapsulation and an atmospheric envelope, redefining the host structure or its void, beyond a singular addition. The visual material indicates that the parasite’s relationship with the host relies on a box-in-box or shell logic that protects or transforms by enclosing it. The Great Court (a) exhibits void wrapping potential; the parasitic roof creates a new interior public space by surrounding the inert courtyard and central structure. Although the roof canopy also rests upon the perimeter walls of the courtyard, the primary action of the intervention is determined as Wrapping, since the enclosure of the void constitutes the most assertive and visually dominant morphological relationship of the case. Cité of Design and Fashion (b) suggests the action’s operation as a second skin; here, the parasite adds a new circulation and identity layer by wrapping the host’s facade. King’s Cross (c) shows how the parasite extends and protects the host structure by wrapping the inert area adjacent to the facade with a massive structural net.

### 4.6. Synthesis: A Morphological Typology

This final synthesis stage of the research marks the transition from the categorical decomposition of the Parasitic Architecture phenomenon to its recomposition as an integrated model. The focus now shifts from the definition of singular morphological actions to the systematic synthesis and integration of the findings derived from them. The collective answers to the fundamental research question indicate that the examined design practices are recurring strategies grounded in the relationship established with the host structure. This finding directly fills the theoretical gap identified in the Theoretical Framework section, specifically the literature’s predominantly descriptive and fragmentary nature, by offering a holistic analytical framework. The matrix presented below (Table 2), which constitutes the analytical backbone of this study, does not merely provide a simple list of the five identified categories; it functions as a holistic analytical tool that indicates the dynamic relationality between the study’s fundamental analytical dimensions. In the schematic diagrams of Table 2, grey represents the host structure, while red indicates the parasitic intervention.

**Table 2:** Morphological typology of parasitic architectural interventions.

Morphological Category (Action)	Host Interface (Contact Surface)	Structural Principle (Attachment Logic)	Spatial Strategy (Spatial Logic)
**Wedging** 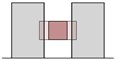	**Opposing Vertical** **Surfaces** Utilizing the void between two buildings or blind facades	**Lateral Bearing/** **Restraint** Bearing, bracing, or suspending between the opposing surfaces	**Volumetric Infill/Patching** Filling and functionalizing a residual urban void
**Perching** 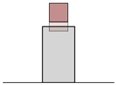	**Upper Horizontal** **Surface** Occupying the roof, terrace, or the apex of the host mass	**Direct Vertical** **Bearing** Resting directly on the host’s exposed upper horizontal surface.	**Superposition/** **Layering** Adding a new independent layer or object atop the mass
**Clinging** 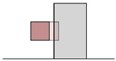	**Single Vertical** **Surface** Attaching to facades, blind walls, or technical piers	**Cantilever/** **Anchored System** Hanging the volume via cantilevers or tensile anchors	**Protrusion/** **Extension** Extending the volume outwardly from the host’s perimeter
**Piercing** 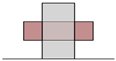	**Shell or Mass** **Integrity** Penetrating the facades, floors, or the host volume itself	**Intersecting/** **Hybrid System** Cutting through the host structure or fusing for balance	**Infiltration/Fusion (Penetration)** Violating boundaries to physically fuse internal spaces
**Wrapping** 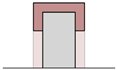	**Volumetric** **Perimeter** Surrounding the entire building, courtyard, or ruin walls	**Independent** **Shell/Cage** Forming a self-supporting envelope around the host	**Encapsulation/** **Enclosure** Enclosing the object within a new atmospheric envelope

**Note:** The “Structural Principle” column describes the attachment logic of each category as documented in the cited sources and observed in the visual material; it is not an engineering analysis of the individual cases.

The findings synthesized in Table 2 relate the Morphological Category to the host interface and to the structural principle described for each category. While it is acknowledged that the link between contact surface and attachment logic is largely governed by mechanical necessity, the framework demonstrates its value by mapping these physical constraints onto specific spatial strategies. The results indicate that the morphological attachment action framework offers an analytical distinction that complements descriptive approaches in the literature, which classify additions solely by location.

The analysis indicates that interventions typically labeled as additions or adaptive reuse operate through a layered operational logic. Table 2 relates the Morphological Category to the host interface and to the structural principle described for each category, without claiming that this relationship is exhaustive. For instance, Wedging extends beyond a mere location between buildings; it links the occupation of the gap between opposing vertical surfaces to the spatial logic of transforming a void into infill. This indicates that, within the Wedging category, the parasite is not merely adjacent to the host but spatially dependent upon it for its existence in the void.

Similarly, the distinction between Perching and Clinging underscores the analytical precision of the typology. While both attach to a host, the table indicates their opposing attachment logic: Perching creates Superposition via Direct Vertical Bearing on an Upper Horizontal Surface, whereas Clinging generates Protrusion through a Cantilever/Anchored System on a Single Vertical Surface. This difference is critical; the former adds a layer atop the mass, while the latter expands its perimeter. In parallel, the Piercing category identifies a more invasive intervention logic, where the parasite targets the Shell or Mass Integrity through an Intersecting System to realize a spatial strategy of Penetration and Fusion. Wrapping, conversely, transcends the addition of a singular object, exemplifying the capacity to redefine the host through Encapsulation by engaging its Volumetric Perimeter.

The primary contribution of this research is the analytical typology proposed for Parasitic Architecture, grounded in the four-axial relationality synthesized in Table 2. Unlike existing studies, this research classifies parasitic structures as morphological and operational systems rather than stylistic or metaphorical entities. Table 2 serves as a categorical definition rather than a mere inventory. The inclusion of the Spatial Strategy column uniquely defines the typology by articulating not only the execution of the action but also its specific urban output, such as Infill, Superposition, Protrusion, Penetration, and Encapsulation.

For example, a rooftop extension is reinterpreted as a Perching typology utilizing direct vertical bearing to achieve superposition, while an inter-building structure represents a Wedging typology generating infill through its occupation of the gap between opposing surfaces. Ultimately, these findings provide a methodological foundation for designers to comprehend the operational logic of interventions on existing stock, opening a pathway through which the parasite can be approached not as an abstract metaphor but as an operational strategy with potential applications in urban densification and adaptive reuse.

It is explicitly acknowledged that the analyzed corpus spans a spectrum with respect to the prototypical criteria of Table 1: while interventions such as the Parasite Office and the Rucksack House embody the temporary and structurally dependent end of this spectrum, others, such as Falkestrasse, the Royal Ontario Museum, or the Great Court, constitute permanent integrations that nevertheless remain parasitic in their morphological relationship to the host. The proposed typology classifies parasitic architecture according to the morphological action performed with respect to the host; it does not measure the degree of parasitism defined by the criteria of Table 1.

## 5. Discussion and Conclusions

This study has grounded the Parasitic Architecture phenomenon, increasingly prevalent in architectural discourse, on an analytical foundation focused on morphological action. The five-category typology matrix (Wedging, Perching, Clinging, Piercing, and Wrapping), synthesized from the corpus analyzed in this study, indicates that parasitic interventions can be systematically organized according to their primary morphological action in relation to the host, as described through the physical interfaces of the host structure, the structural principle derived from that interface, and the spatial strategies of the parasite.

The five categories derived from the corpus and presented in Table 2 classify the spatial and morphological dimensions of this relationship, indicating parallels with specific biological attachment classifications. Within this context, Wedging finds its closest biological counterpart in coelozoic dwelling, where the organism inhabits existing host cavities [55]. Perching corresponds to epiphytism, a commensal relationship utilizing the host solely for structural support [56]. Clinging is paralleled by ectoparasitic adhesion, in which organisms secure themselves to the external surface using dedicated attachment organs, such as a haptor or scolex [57,58]. Piercing aligns with mesoparasitic attachment, utilizing penetrating structures like a haustorium to partially embed into the host tissue [59,60]. Finally, wrapping reflects the behavior of hemiepiphytes and twining parasites, which establish their existence by completely enveloping and encapsulating the host [61,62].

The theoretical contribution of the study can be assessed against existing classification models. Approaches grounded in Onur [25] and Rush [26], and synthesized by researchers like Zakar [27], classify additions primarily through static location (horizontal/vertical) and geometric relation (adjacent/detached). While offering a valuable reference system for heritage integration, these static definitions remain insufficient to explain the dynamic attachment logic required by parasitic architecture. For instance, a structure definable simply as Vertical + Adjacent in traditional literature diverges into two distinct strategies in this action-based typology: Perching (direct vertical bearing) or Clinging (lateral attachment).

The findings engage in a robust dialogue with international adaptive reuse theories. While Wong [28] broadly defines parasitic interventions as a strategy of addition, this study operationalizes this general definition by decomposing it into five specific morphological actions. The proposed typology can also be read through Brand’s [29] shearing layers approach, which indicates its analytical precision. According to this typology, Wrapping constitutes an intervention targeting the skin layer; Piercing constitutes a radical violation of the structure layer; Wedging constitutes an intervention at the scale of the site, occupying a residual void between existing masses; Perching and Clinging both constitute interventions targeting the skin layer, that is, the envelope. Perching rests on its upper surface without attachment, while Clinging fixes itself to its vertical surface through a dedicated anchoring device. The comparison indicates that the typology distinguishes not only between layers but also between modes of engagement within a single layer. The comparison is offered as an interpretive reading rather than a direct correspondence, since the shearing layers are defined by their different rates of change [63] and describe the physical composition of buildings rather than design and spatial aspects [64]. Wedging is the most interpretive of the five, since its association with the site layer follows from the position of the cases within interstices between existing masses rather than from a documented engagement with the site. Consequently, this research contributes to the literature by detailing Machado’s [30] aesthetic-oriented remodeling definitions with a morphological and spatial logic of attachment.

Beyond these adaptive reuse readings, a second line of comparison is offered by operational frameworks that classify spatial interventions through action. Kamalipour and Dovey [6] decompose the incremental production of urban space into actions such as extend, attach, replace, divide, infill, and connect; Robert’s [5] relational scheme of building over, within, alongside, and around offers a comparable vocabulary of operations; and Given [7] classifies parasitic forms according to the nature of the host relationship rather than its location. However, these frameworks do not address the host interface through which each action is physically realized, which is precisely the dimension contributed by the morphological typology proposed in this study. Read together with the comparison to static classifications above, this locates the contribution of the study between the two existing lines: whereas the static models describe position and the action-based models name the operation, the proposed typology specifies the physical attachment action together with its host interface, structural principle, and spatial strategy. The typology also reconnects these findings to the urban morphology literature. Viewed through the Conzenian triad of street, plot, and building, Wedging appears as an acceleration of incremental infilling processes, while Perching constitutes a discrete episode of volumetric growth upon the building block. This interpretation aligns with Kostourou’s [65] longitudinal work mapping volumetric transformations, which indicates that such additions represent a normal process of urban morphogenesis rather than an anomaly. The five categories thus operate at the intersection defined as typomorphology: they extend the morphological reading of the existing stock to the attachment actions performed upon it. The remaining categories act at a finer register: Clinging and Wrapping engage the envelope of the existing building rather than the tissue, and Piercing its internal structure, which is why they are read through the layer comparison above.

The Perching category also connects to a broader body of European literature on vertical densification and roof stacking. Methodological mapping studies, comprehensive reviews, and case evaluations highlight rooftop extensions as a key strategy for urban densification. Broader technical, regulatory, and modular studies across Europe similarly conceptualize these additions as holistic morphological operations that generate a new urban layer above the existing city [66,67]. However, the Perching category within this framework diverges from these conventional vertical additions, which tend to prioritize structural and legal integration, by defining the intervention as an independent and alien object relative to the host. The distinction lies in the object of the intervention: conventional vertical additions seek integration with the existing structure, whereas Perching maintains an alien position by design.

Furthermore, the study offers an operational grammar that connects theoretical discourses treating the parasite merely as a philosophical metaphor [10] or a political gesture [23] with practical architectural production, indicating a promising direction for the application of the typology in design. Analyzed cases, such as narrow lot infills in the Wedging category or industrial transformations in the Piercing category, indicate that the parasitic action is not merely an invasion but an infill and fusion strategy activating urban voids and the residual potentials of existing structures.

The analytical flexibility of the proposed typology is apparent in cases whose classification is not immediately evident and which remain unresolved in conventional models. For instance, projects disguised as rooftop technical volumes (e.g., H-VAC Antepavilion) are identified here as a sub-manifestation of the Perching category. The case is considered to illustrate a mimetic disguise [68]: the parasitic intervention adopts the appearance of an inert infrastructural element, a strategy parallel to masquerade in biological systems, in which organisms resemble inanimate objects to avoid detection [69]. Similarly, projects enveloping the existing structure (e.g., Cité of Design and Fashion) indicate that the parasitic action operates not merely as an addition but as a second skin strategy redefining the existing structure.

The limits of the proposed typology become apparent in cases where the documentation does not support a single dominant morphological action. Two cases examined in this study mark these limits. In the case of Keret House, no mechanical load path between the intervention and the host structure is documented in the available material, and the classification of the case within the Wedging category therefore rests on the morphological position of the volume within the void rather than on a verified structural engagement. In the case of the Royal Ontario Museum, several morphological actions are combined within a single intervention, and the assignment to one category required the identification of the primary action. Neither case invalidates the proposed classification; both indicate that the typology is most operative where a single morphological action is dominant and legible in the documentation of the case.

In conclusion, this research repositions Parasitic Architecture within the context of urban densification, heritage conservation, and the reuse of residual spaces. Within the exploratory scope of this study, the developed morphological typology offers a common language and analytical tool for architects, urban planners, and researchers to understand, design, and critique these interventions, and thereby constitutes a first step toward addressing the theoretical gap identified in this study. In this framework, parasitic architecture is reframed not as a virus but as a potentially symbiotic urban strategy, whose capacity to update the existing urban fabric functionally and aesthetically is a proposition of this study rather than a demonstrated outcome. The framework is exploratory in nature: since the typology is derived from fifteen documented cases, with three cases per category, its categories are proposed as analytical instruments to be tested rather than as a validated classification, and its application beyond this corpus remains to be tested.

## 6. Recommendations

The morphological typology and the analytical matrix (Table 2) introduced by this research are proposed as a step from a descriptive plane toward the level of an operational design strategy. As an exploratory framework derived from fifteen documented cases, it is intended not only to classify existing literature but also to offer a provisional basis for guiding future forms of urban production. Considering global crises such as urban densification, sustainability, and the re-evaluation of existing building stock, parasitic architecture could, under conditions that remain to be examined, evolve from a niche production into a more widely applied strategy. Assessing whether such a transformation occurs and testing the transferability of the analytical model beyond the analyzed corpus will require new perspectives that expand the research boundaries.

Accordingly, it is primarily necessary to test the proposed framework, the scope of which, as stated in the Methods section, is limited to documented cases located in European and North American contexts, in different geographical and socio-economic contexts. Future research should critically test this proposed morphological typology on informal settlements and spontaneous, vernacular additions. Examining how actions like Clinging or Wedging manifest in architecture without architects, regions with flexible regulations, or post-crisis housing solutions would test whether the typology can operate beyond a stylistic analysis tool and function as a transferable housing strategy. Such comparative studies would be required to strengthen the external validity of the typology and expand the model’s cultural boundaries.

Beyond academic research, utilizing the matrix developed by the study as an active pedagogical tool in architectural education, especially in adaptive reuse studios, is recommended. This typology offers students and designers the opportunity to transcend general concepts like making an addition when intervening in an existing structure. Encouraging students during the design process to think in an action-oriented manner, such as developing a clinging strategy using the blind facade or constructing a wedging action using the void between two buildings, can strengthen the structural and spatial consistency of the design. In this context, the matrix is proposed as a generative design methodology, not only to analyze what exists but also to open the possibility of producing new and contextual parasitic interventions.

Finally, extending the definition of the host, which this study limited to the building scale, to the urban infrastructure scale could reframe the role of parasitic architecture in urban transformation. Actions like Piercing and Wrapping hold great potential not only on buildings but also on urban voids left inert by urban infrastructures such as under-bridges, viaducts, metro stations, and highway verges. It is suggested that future studies adapt this typology to urban infrastructure projects, where it can serve as a strategy guide for improving urban voids and reintegrating them into the public realm. Thus, the parasitic action could, in principle, evolve from being a singular building addition into an infrastructural layer capable of repairing the urban fabric from within; whether this potential is realized in practice remains to be demonstrated. The extensions outlined above are offered as potential applications of the typology, indicating directions for its further development in urban design and adaptive reuse.

## Figures and Tables

**Table 1:** Conceptual comparison between conventional addition and parasitic architecture.

Criteria	Conventional Addition	Parasitic Architecture
Dependency & Exploitation	Possesses independent structural integrity, relying on the main building primarily for connection while respecting its stability.	Tends to rely on the host for spatial or physical support, exploiting its structure or infrastructure on a facultative-to-obligate spectrum.
Nature of Relationship	Establishes a harmonious or complementary dialogue, aiming to complete the host’s form through respectful continuity.	Typically establishes a tense or provocative dialogue to challenge the host’s identity through an act of invasive contrast.
Temporariness & Reversibility	Designed as a permanent and static integration, physically merging with the host to become an inseparable part of the whole.	Typically designed as a temporary or detachable addition perching on the host, though some interventions constitute permanent integrations.
Motivation & Strategic Intent	Driven by pragmatic functional needs, primarily aiming to expand the available floor area or accommodate specific uses.	Often driven by critical or political intent, primarily aiming to highlight urban voids or question the existing spatial norms.

**Note:** The characteristics listed under “Parasitic Architecture” describe the prototypical parasitic condition. The cases included in the corpus exhibit these features to varying degrees; the classification of cases in this study is based on the morphological action performed with respect to the host, not on the degree to which each criterion is satisfied.

## Data Availability

The data presented in this study are available in the article.

## Appendix A

**Table A1:** Complete list of the analyzed parasitic architectural interventions.

Project Name	Architect/Firm	Year	Location	Source	Host Interface	Category
Parasite Office	Za Bor Architects	2011	Moscow/Russia	[40]	Opposing Vertical Surfaces	Wedging
Stadtmuseum	MLZD	2012	Rapperswil-Jona/Switzerland	[41]		
Keret House	Centrala	2012	Warsaw/Poland	[42]		
Remodeling Falkestrasse	Coop Himmelb(l)au	1988	Wien/Austria	[43]	Upper Horizontal Surface	Perching
The Cube Restaurant	Park Associati	2011	Milan/Italy	[44]		
H-VAC Antepavilion	PUP Architects	2017	London/UK	[45]		
Rucksack House	Stefan Eberstadt	2004	Leipzig/Germany	[46]	Single Vertical Surface	Clinging
Convent de Sant Francesc	David Closes	2011	Santpedor/Spain	[47]		
Clip-On Centraal Museum	Atelier van Lieshout	1997	Utrecht/Netherlands	[48]		
Royal Ontario Museum	Daniel Libeskind	2007	Toronto/Canada	[49]	Shell or Mass Integrity	Piercing
Fortress of Franzensfeste	Markus Scherer	2009	Fortezza/Italy	[50]		
Bombay Sapphire Distillery	Heatherwick Studio	2014	Hampshire/UK	[51]		
The Great Court	Foster + Partners	2000	London/UK	[52]	Volumetric Perimeter	Wrapping
Cité of Design and Fashion	Jakob + MacFarlane	2008	Paris/France	[53]		
King’s Cross Station	John McAslan + Partners	2012	London/UK	[54]		
